# Selective Transport of Protein-Bound Uremic Toxins in Erythrocytes

**DOI:** 10.3390/toxins11070385

**Published:** 2019-07-01

**Authors:** Olivier Deltombe, Griet Glorieux, Sami Marzouki, Rosalinde Masereeuw, Daniel Schneditz, Sunny Eloot

**Affiliations:** 1 Department of Internal Medicine and Pediatrics, Nephrology Section, Ghent University Hospital, 9000 Ghent, Belgium; 2 Department of Pharmaceutical Sciences, Pharmacology, Utrecht University, 3584 CG Utrecht, The Netherlands; 3 Otto Loewi Research Center, Physiology, Medical University of Graz, 8010 Graz, Austria

**Keywords:** protein-bound uremic toxins, hippuric acid, indole-3-acetic acid, indoxyl sulfate, *p*-cresyl sulfate, erythrocyte, DIDS, MK571, KO143, hemodialysis, chronic kidney disease

## Abstract

To better understand the kinetics of protein-bound uremic toxins (PBUTs) during hemodialysis (HD), we investigated the distribution of hippuric acid (HA), indole-3-acetic acid (IAA), indoxyl sulfate (IS), and *p*-cresyl sulfate (*p*CS) in erythrocytes of HD patients. Their transport across the erythrocyte membrane was explored in the absence of plasma proteins in vitro in a series of loading and unloading experiments of erythrocytes from healthy subjects and HD patients, respectively. Furthermore, the impact of three inhibitors of active transport proteins in erythrocytes was studied. The four PBUTs accumulated in erythrocytes from HD patients. From loading and unloading experiments, it was found that (i) the rate of transport was dependent on the studied PBUT and increased in the following sequence: HA < IS < *p*CS < IAA and (ii) the solute partition of intra- to extra-cellular concentrations was uneven at equilibrium. Finally, inhibiting especially Band 3 proteins affected the transport of HA (both in loading and unloading), and of IS and *p*CS (loading). By exploring erythrocyte transmembrane transport of PBUTs, their kinetics can be better understood, and new strategies to improve their dialytic removal can be developed.

## 1. Introduction

The group of protein-bound uremic toxins (PBUTs) include all uremic retention solutes binding to plasma proteins with most of them having a molecular weight <500 Da [1,2]. The percentage that is bound to proteins (% protein binding, % PB) is dependent on the solute itself rather than on binding competition or protein saturation [3] and may reach more than 99% [4,5]. In normal conditions, the free (i.e., unbound) fraction of the toxins is passively cleared by glomerular filtration, whereas organic anion and cation transporters expressed on renal proximal tubule cells are responsible for the active secretion of the protein-bound fraction into the urine [6,7].

In patients with end-stage kidney disease treated with hemodialysis (HD), only the fraction that is not bound to proteins can pass the pores of the hemodialyzer membrane. Consequently, the dialyzer clearance of these solutes is much lower than that for comparable small non-protein-bound solutes, especially when the % PB is high [8,9,10]. During the past decade, studies have been performed aiming to improve the removal of these PBUTs during hemodialysis. Amongst others, the removal of PBUTs was shown to be enhanced when using extended HD and hemodiafiltration [11,12], by changing the local ionic strength at the blood inlet of the dialyzer increasing the free fraction [13], and by combining dialysis with adsorption [14,15,16,17]. Beside the studies on extended HD, most of these papers focused only on the removal of PBUTs from the plasma compartment, ignoring the role of erythrocytes constituting almost 30–40% of the blood volume passing the dialyzer [10,18]. 

Small water-soluble solutes are known to distribute in plasma and erythrocytes [19] and the transport through the erythrocyte membrane has been studied before for urea, uric acid, and creatinine [20,21,22,23,24]. For PBUTs, the distribution in erythrocytes as well as the transport through the erythrocyte membrane is unknown, but may help to explain their kinetics in the patient [5,9] as well as in the hemodialyzer [5].

To address this question, the present study investigated the distribution of four different anionic PBUTs (hippuric acid (HA), indole-3-acetic acid (IAA), indoxyl sulfate (IS), and *p*-cresyl sulfate (*p*CS)) in erythrocytes as well as their transport across the erythrocyte membrane. Furthermore, to better understand the transport mechanism across the erythrocyte membrane, the impact of different transporter protein inhibitors was studied: i.e., the inhibitor DIDS (4,4′ diisothiocyanato-2,2′-stilbenedisulfonic acid) of band 3 anion transporters, MK571 (L66071 sodium salt) of MRP1 (multidrug resistance-associated protein 1), and KO143 of BCRP (breast cancer resistance protein; also known as ATP-binding cassette transporter G2 - ABCG2) [25,26,27,28,29,30].

## 2. Results

### 2.1. Presence of Protein-Bound Uremic Toxins (PBUTs) in Erythrocytes

In predialysis blood from HD patients, it appeared that HA, IAA, IS, and *p*CS are present in erythrocytes, as determined by their concentration in the cell pellets viz. 88.1 (22.0; 239.1), 2.4 (1.8; 2.9), 29.7 (15.9; 33.9), and 25.8 (18.4; 30.6) µmol/L, respectively.

### 2.2. Experimental Data for Loading and Unloading Experiments

For each series of experiments, PBUT concentrations were determined in the buffer (phosphate buffered saline (PBS) or Hank’s Balanced Salt Solution (HBSS)/ hydroxyethyl piperazineethanesulfonic acid (HEPES) for loading and unloading, respectively) supernatant, calculated immediately after spiking (C_BUFFER(0)_,c) (loading) or being zero (unloading), and measured at equilibrium (C_BUFFER(eq)_,m) in the absence and presence of transporter protein inhibitors (Table 1). In addition, hematocrit in a native whole-blood sample (H_wb_) and hematocrit in erythrocyte suspension (H_susp_) are provided in Table 1.

### 2.3. Transport across the Erythrocyte Membrane—Influx

**Figure 1:**
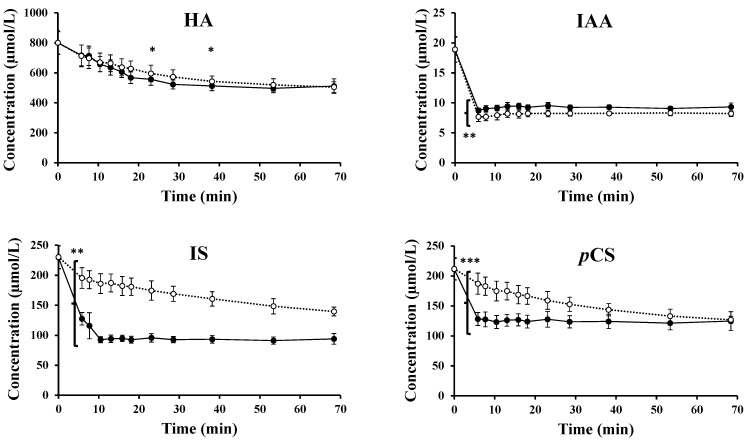
Mean concentration over time for hippuric acid (HA), indole-3-acetic acid (IAA), indoxyl sulfate (IS), and *p*-cresyl sulfate (*p*CS), as measured in the PBS fraction during loading experiments in the absence (black dots and full line) and presence (white dots and dotted line) of the inhibitor DIDS (4,4′ diisothiocyanato-2,2′-stilbenedisulfonic acid), respectively. Within each condition, the decrease in supernatant PBUT concentration was significant up to 38 min (HA), up to 10 min (IAA and IS in the absence of the inhibitor), or 38 min (IAA, IS, and *p*CS in the presence of the inhibitor) respectively, but is not indicated on the figures for clarity. * *p* < 0.05 versus absence of the inhibitor at the same time points; ** *p* < 0.05 versus absence of the inhibitor during the complete time course; *** *p* < 0.05 versus absence of the inhibitor up to 38 min. All *n* = 8.

The decrease in mean HA, IAA, IS, and *p*CS concentration in the PBS fraction over time, as obtained in loading experiments of erythrocytes from healthy subjects in absence of an anion transporter protein inhibitor (DIDS), is illustrated in Figure 1 (black dots and full line). After 6 min, this decrease was significant, as compared to the theoretical start concentration for all four PBUTs, and the concentrations further decreased to reach an equilibrium at 38 min for HA and 10 min for IAA and IS. For *p*CS, the concentration decreased almost immediately after spiking and an equilibrium was already reached at the first experimental time point. 

In the presence of the transporter protein inhibitor, DIDS, the toxin influx was affected by the change in mean HA, IAA, IS, and *p*CS concentration in the PBS fraction over time, as illustrated in Figure 1 (white dots and dotted line). Here again, concentrations of the four PBUTs decreased over time and reached an equilibrium at 38 min for HA. For IAA, IS, and *p*CS however, no equilibrium was formed within the time course of the experiment, indicating a slower transport in the presence of the inhibitor.

The impact of DIDS is visible in the PBUT concentrations remaining lower (i.e., for IAA) and higher (i.e., for IS) in the PBS during the complete experimental time course, and for *p*CS at least up to 38 min (Figure 1). Overall, HA transport was only slightly influenced by the presence of DIDS with concentrations significantly different at 25 and 40 min.

### 2.4. Transport Across the Erythrocyte Membrane—Efflux

**Figure 2:**
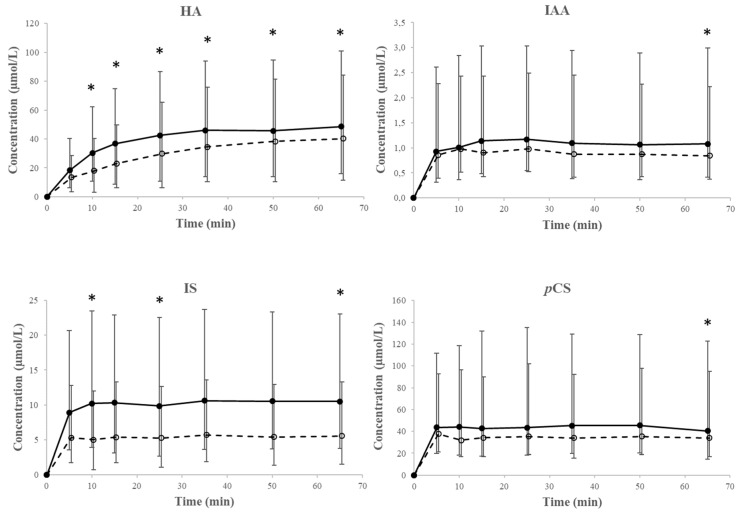
Median concentrations over time (with 25th and 75th percentile as error bars) for hippuric acid (HA), indole-3-acetic acid (IAA), indoxyl sulfate (IS), and *p*-cresyl sulfate (*p*CS), as measured in the HBSS/HEPES during unloading experiments in the absence (black dots and full line) and presence (white dots and dotted line) of the inhibitors DIDS, MK571, and KO143. * *p* < 0.05 versus absence of the inhibitor at the same time points. All *n* = 6.

Figure 2 shows the median HA, IAA, IS, and *p*CS concentrations (and 25th and 75th percentiles) measured in the HBSS/HEPES, as obtained during the unloading experiments of erythrocytes from HD patients (black dots and full line). Despite the large inter-patient variability, a trend in increasing HA concentration was observed. HA concentrations measured in the HBSS/HEPES were found to be significantly increased already after 10 min, as compared to the concentration at *t* = 0 min, and remained unchanged from 38 min onward (Figure 2). For IAA, IS, and *p*CS however, this transport was very fast and corresponding concentrations were already in equilibrium at the first experimental time point.

In the presence of the inhibitors of protein transporters, DIDS, MK571, and KO143, toxin efflux was affected for HA and in a lesser degree for IS (Figure 2). While equilibrium was formed very fast for IS, *p*CS, and IAA, no equilibrium was formed within the time course of the experiment for HA.

### 2.5. Kinetic Analysis 

Experimental data from all loading and unloading experiments were used to fit the following kinetic parameters for HA, IAA, IS, and *p*CS: the equilibration time constant (a), specific rate constant (k_s_), intercompartment clearance (K_C_) and, for the loading experiment without DIDS, also the solute partition coefficient (γ) (Table 2).

In loading experiments without the inhibitor, parameters a, k_s_, and K_C_ were found to be the lowest for HA, followed by those for IS (trend only), *p*CS, and IAA. 

In loading experiments in the presence of an inhibitor, the transport parameters a, k_s_, and K_C_ reached comparable low values for HA, IS, and *p*CS, whereas those for IAA were not affected (Table 2).

In unloading experiments, both without and with inhibitors, kinetic parameters a, k_s_, and K_C_ were significantly higher for IAA, IS, and *p*CS, as compared to those for HA. No differences were found for the kinetic parameters in the experiments without versus with inhibitors.

## 3. Discussion

In this work, the intracellular concentration of PBUTs in erythrocytes of HD patients was measured and the transport of these solutes across the erythrocyte membrane was studied in vitro in blood (influx) from healthy controls as well as in blood from HD patients (efflux). Furthermore, a first attempt was undertaken to determine the transport mechanism of these PBUTs across the erythrocyte membrane by using different transporter protein inhibitors, i.e., DIDS, MK571, and KO143. The main findings were: (i) HA, IAA, IS, and *p*CS are distributed in erythrocytes; (ii) the rate of transport (i.e., both influx and efflux) is dependent on the studied PBUT and increases in the following sequence: HA < IS < *p*CS < IAA; (iii) the presence of DIDS, which inhibits anion transport via Band 3 protein across the erythrocyte membrane, slows down the uptake of HA, IS, and *p*CS in the erythrocyte, and (iv) the presence of DIDS, MK571, and KO143 seems to impact HA and, although less expressed, IS efflux, but has no substantial impact on the efflux of IAA and PCS. 

The PBUTs studied distribute within the erythrocytes, which was confirmed by spiking an erythrocyte suspension with a high uremic concentration of PBUTs and measuring their uptake over time. After PBUT addition to the extracellular (PBS) compartment, concentrations in PBS decreased either slowly (i.e., for HA), at intermediate speed (i.e., for IS), or very fast (i.e., for *p*CS and IAA) so that an apparent equilibrium between the PBS and erythrocyte compartments was established after respectively, 38 min, 10 min, or almost immediately. 

Obtained transport parameters (i.e., equilibration time constant (a), specific rate constant (k_s_), and intercompartment clearance (K_C_)) demonstrated that HA is more slowly transported into erythrocytes as compared to IS (trend), *p*CS, and IAA. When unloading erythrocytes from HD patients suspended in HBSS/HEPES, and based on the obtained kinetic parameters, it was found that the rate of solute efflux was comparable to the rate of solute influx for IAA, but higher for HA and IS while lower for *p*CS.

It appeared that the influx and efflux of IAA across the erythrocyte cell membrane is much faster than for HA and IS, which is reflected by the high values for a, k_s_, and K_C_. Concentrations of indole, the in vivo precursor of IAA, were determined in some of the loading experiment samples to check whether the fast decrease in IAA concentrations was due to a fast back transformation of IAA into indole. However, indole concentrations were negligible in PBS (data not shown). Alternatively, (part of) IAA could bind to the erythrocyte membrane surface or to proteins present in the cell membrane immediately after addition of the PBUT mix, resulting in an apparent fast removal from the PBS compartment. However, to the best of our knowledge, no data of IAA binding to erythrocyte membranes is available in the literature to support this hypothesis.

We also studied the impact of an inhibitor (i.e., DIDS) for anion transport. This compound is known to reversibly bind to Band 3 proteins, anion exchangers located on erythrocyte membranes, mediating transmembrane transport [25,26,27,28]. Because these Band 3 proteins can only influence solute transport, the distribution of PBUTs will not be changed. Hence, in the kinetic model of the loading experiments with DIDS, solute partition coefficients can be taken as equal to those found in the loading experiments without DIDS. By doing this, we observed that the transport parameters a, k_s_, and K_C_ decreased to comparable low values for HA, IS, and *p*CS. For this reason, the transport of HA, IS, and *p*CS is at least in part facilitated by Band 3 proteins (i.e., carrier-mediated facilitated diffusion). 

Notwithstanding the similar equilibration time constants for HA, i.e., 0.06–0.10 1/min, as found in the present work and those for creatinine, i.e., 0.05 ± 0.01 1/min, as previously reported by Schneditz et al. [23], the transport mechanism of both compounds (and partly for IS and *p*CS as well) might not be comparable. This is mainly because of the different net charge of these compounds at pH 7.4 (i.e., positive for creatinine and negative for HA, IS, and *p*CS), although similarities between their renal handling, also affecting their plasma levels, has been reported [31]. Apart from the anion exchanger Band 3, other anion transporter proteins may also be involved in the transport of the studied PBUTs. It has been shown that the multidrug resistance protein 1 (MRP 1) is expressed by erythrocyte membranes [32,33]. This transporter is known to efflux several sulfate conjugates of endogenous as well as of xenobiotic compounds from diverse tissues [34,35] and might potentially be involved in the transmembrane transport of IS and *p*CS as well. More recently, a database (http://rbcc.hegelab.org/) was created containing information on other different transport proteins present in the erythrocyte membrane [36]. These include the ATP Binding Cassette efflux pumps MRP4 and BCRP, in addition to MRP1. Since a combination of blockers was used in the unloading experiments with overlapping potencies, we expect, however, efficient inhibition of these transporters in our experimental settings [37]. 

The distribution of solutes within erythrocytes and the slow transport from erythrocytes to plasma has important consequences for their removal during hemodialysis. For example, for solutes slowly equilibrating across the erythrocyte membrane, the true concentration in plasma leaving the hemodialyzer is much lower than what is measured when solutes are primarily removed from plasma, and intracellular solutes remain sequestered in erythrocytes. The concentration measured in a plasma sample, however, depends on the time the blood sample is allowed to equilibrate (i.e., the time erythrocytes are allowed to “unload” their solutes) and on the rate of solute equilibration between plasma and erythrocytes before the blood components are separated by centrifugation. If blood is collected at the dialyzer outlet line, there is a disequilibrium between the intra- and extra-cellular concentrations. For solutes fast equilibrating across the erythrocyte membrane, the determined solute plasma concentration could be higher, as it really was at the moment of collection, which would falsely underestimate extracorporeal solute clearance, as previously demonstrated for creatinine [38]. For the present PBUTs, we also found that the time between blood sampling and centrifugation affects the serum/plasma concentration in samples collected at the dialyzer outlet line (data provided in Appendix A).

Beside the % PB, the magnitude and rate of accumulation of PBUTs in erythrocytes may also have an impact on the amount of solute removed by the dialyzer. For solutes rapidly equilibrating across the erythrocyte membrane, solute is not only cleared from the plasma compartment but also from the erythrocyte compartment, and plasma and erythrocyte concentrations will be close to equilibrium at the dialyzer inlet as well as at the outlet. On the contrary, for solutes slowly equilibrating across the erythrocyte membrane, such as creatinine, the transport across the membrane should be taken into account, as described elsewhere [39]. The exact fraction of extracorporeal blood flow cleared in the dialyzer can be estimated from dialyzer transit time, hematocrit, and specific rate constant [23], but should be adjusted by a solute partition coefficient for the studied PBUTs.

## 4. Conclusions

This is the first study to identify and quantify intracellular distribution of PBUTs in erythrocytes. The rate of transport (i.e., influx and efflux) across the erythrocyte membrane increased according to HA < IS < *p*CS < IAA. In addition, at least part of the HA, IS, and *p*CS uptake by erythrocytes is attributed to the anion transporter Band 3 protein. Understanding distribution and transport in the patient’s body, including transcellular transport in blood, is of importance to be able to optimize dialysis treatments with eventual newly-developed removal strategies.

## 5. Materials and Methods 

### 5.1. Sample Collection

Blood samples from 6 HD patients were collected predialysis to determine the presence of PBUTs in erythrocytes, while blood was sampled from 8 healthy controls and another 6 HD patients (predialysis) to be used in the loading and unloading experiments, respectively. All blood was sampled in K_2_EDTA plasma tubes (Becton Dickinson, Plymouth, UK). 

This study was conducted according to the Declarations of Helsinki, was approved by the Ethics Committee of Ghent University Hospital (2017/0162), and all participants gave their written informed consent.

### 5.2. Chemicals

HA, IAA, and IS were purchased from Sigma-Aldrich (St. Louis, MO, USA) and *p*CS was obtained from TCI Chemicals (Zwijndrecht, Belgium). Water (HPLC grade) was purchased from Acros Organics (Thermo Fisher Scientific, Geel, Belgium), the inhibitor DIDS from Sanbio (Cayman Chemical, MI, USA), and the inhibitors MK571 and KO143 from Sigma-Aldrich (Saint Louis, MO, USA). 

For the loading experiments, a stock solution containing HA, IAA, IS, and *p*CS (further assigned as PBUT mix) as well as a DIDS stock solution were both prepared in phosphate buffered saline (PBS) buffer pH = 7.4 (Invitrogen, Thermo Fischer Scientific, Ghent, Belgium) and stored at −20 °C. Theoretical final PBUT concentrations were based on the maximum uremic levels as reported by Duranton et al. [2]. Therefore, stock concentrations (200 times the final concentration) of 80 mmol/L (HA), 2 mmol/L (IAA), and 20 mmol/L (IS and *p*CS) were prepared. For DIDS, a stock solution of 1300 µmol/L (13 times the final concentration) was prepared.

For the unloading experiments, additional stock solutions of the inhibitors MK571 and KO143 were prepared in Hank’s Balanced Salt Solution (HBSS) with 10 mmol/L HEPES (4-(2-hydroxyethyl)-1-piperazine-ethanesulfonic acid) (StemCell, Vancouver, Canada) at a concentration of 1000 µmol/L and 5,000 µmol/L, respectively.

### 5.3. In Vitro Protocols

In general, to separate erythrocytes and plasma, blood was centrifuged for 10 min at 2095 *g*, at room temperature (Beckman Coulter X-15R centrifuge-VWR, Leuven, Belgium).

#### 5.3.1. Proof of Concept—Presence of PBUTs in Erythrocytes?

After centrifugation of blood from HD patients, plasma was removed and PBUT concentrations were determined in the erythrocyte pellet. This pellet was lysed during the first step of the sample preparation where HPLC grade water was added, followed by heating up to 95 °C.

#### 5.3.2. Transport of PBUTs across the Erythrocyte Membrane

In loading (i.e., influx) experiments, after centrifugation of blood from healthy controls, plasma from 13 mL blood samples was replaced by an equal volume of PBS in the absence (PBS) and the presence of the Band 3 protein blocker DIDS (final concentration: 100 µM, aiming at a complete inhibition of the influx of PBUTs via the transporter protein Band 3 without exerting cytotoxic effects). The obtained erythrocyte suspension was gently mixed and pre-incubated in a water bath at 37 °C for 1 h. Subsequently, a PBUT mix stock solution (final concentrations: 400 µM HA, 10 µM IAA, and 100 µM IS and *p*CS) was added and the suspension was gently mixed. 

In the unloading experiments, 13 mL of whole blood from HD patients was pre-incubated for 10 min at 37 °C in the absence (HBSS/HEPES) and the presence of 3 transporter inhibitors (final concentrations: 50 µM DIDS, 3 µM MK571, and 5 µM KO143). Next, whole blood was centrifuged, and the plasma was replaced by an equal volume of HBSS/HEPES again in the absence and the presence of the transporter inhibitors (final concentration: 80 µM DIDS, 5 µM MK571, and 8 µM KO143, aiming at a complete inhibition of the efflux of PBUTs via the transporter proteins Band 3, MRP1, and BCRP without exerting cytotoxic effects). 

After gently mixing the erythrocyte suspension in each series of experiments, aliquots (1 mL) were incubated in a water bath at 37 °C while continuously shaken to avoid erythrocyte sedimentation. At certain specific time points, an aliquot was removed from the water bath, immediately centrifuged (Beckman Coulter Microfuge 18–Analis, Ghent, Belgium, 2,306 g, 5 min), and the obtained supernatant and pellet was stored at −80 °C until batch analysis.

### 5.4. Analyses

Total PBUT concentrations were determined by an ultra-high-performance liquid chromatography instrument with ultraviolet (for HA) and fluorescence (for IAA, IS, and *p*CS) detection (UHPLC-UV/FLD). Both sample preparation and chromatographic methods were previously described in more detail [40]. 

Hematocrit (H) was determined by transferring blood into capillary tubes before centrifugation (Hettich centrifuge—Tuttlingen, Germany, 9503 g, 2 min) and was manually read on a calibrated plate in all loading and unloading experiments, in the non-treated whole blood samples as well as in the corresponding erythrocyte suspensions.

### 5.5. Kinetic Model

**Figure 3:**
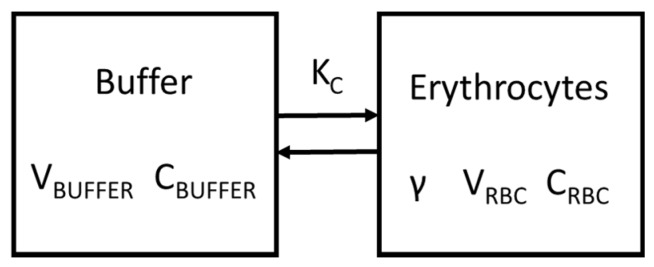
Two-compartment model. V_BUFFER_: Buffer water volume; C_BUFFER_: solute concentration in buffer water volume; γ: solute partition coefficient; V_RBC_: erythrocyte water volume; C_RBC_: solute concentration in erythrocyte water volume; K_C_: intercompartment clearance.

The transport characteristics for a solute evenly equilibrating across intra- and extra-cellular compartments has been described using a two-compartment model [23]. This model, however, needs to be adapted for solutes with non-uniform equilibration, as schematically shown in Figure 3. Differences in distribution can be quantified by the solute partition coefficient (γ) defined as the ratio of solute concentrations in both compartments at equilibrium [41]. In this work, those two compartments included erythrocytes (red blood cells, RBC: C_RBC_) and BUFFER (C_BUFFER_):

(1) $$\gamma \;=\;\frac{C_{\mathrm{RBC}}}{C_{\mathrm{BUFFER}}}$$

Assuming constant erythrocyte and buffer volumes, the two-compartment model for solute equilibration in a blood sample between erythrocytes and buffer is given as:

(2) $$\begin{array}{l} V_{\mathrm{BUFFER}}\;\frac{{\mathrm{dC}}_{\mathrm{BUFFER}}}{\mathrm{dt}}{=K}_{C}\;\left(\frac{C_{\mathrm{RBC}\;}}{\gamma }{-C}_{\mathrm{BUFFER}}\right)\\ V_{\mathrm{RBC}}\;\frac{{\mathrm{dC}}_{\mathrm{RBC}}}{\mathrm{dt}}{=-K}_{C}\;\left(\frac{C_{\mathrm{RBC}\;}}{\gamma }{-\;C}_{\mathrm{BUFFER}}\right) \end{array}$$

where, V_BUFFER_ and V_RBC_ (both L) refer to the volumes of buffer water and erythrocyte water (i.e., the cytoplasm), C_BUFFER_ and C_RBC_ (both mol/L) are PBUT concentrations in buffer and erythrocyte compartments respectively, γ accounts for intracellular partition, and K_C_ (L/min) refers to the intercompartment clearance.

The total mole n (in mol) of solute in the entire erythrocyte suspension is constant:

(3) $${n=V}_{\mathrm{BUFFER}}C_{\mathrm{BUFFER}}{+V}_{\mathrm{RBC}}C_{\mathrm{RBC}}$$

so that Equation (2) can be simplified to obtain the following relationship:

(4) $$\frac{{\mathrm{dC}}_{\mathrm{BUFFER}}}{\mathrm{dt}}=\frac{K_{C}n}{{\gamma V}_{\mathrm{RBC}}V_{\mathrm{BUFFER}}}-\frac{K_{C}\;\left({\gamma V}_{\mathrm{RBC}}{+V}_{\mathrm{BUFFER}}\right)}{{\gamma V}_{\mathrm{RBC}}V_{\mathrm{BUFFFER}}}C_{\mathrm{RBC}}$$

The solution of this ordinary differential equation for the interval t = 0 to t = t is:

(5) $$C_{\mathrm{BUFFER}(t)}=\left(C_{\mathrm{BUFFER}(0)}-\frac{b}{a}\right)e^{-\mathrm{at}}-\frac{b}{a}$$

where, the macro parameters b (in mol/min/L) and equilibration time constant a (in 1/min) are given as:

(6) $$b=\frac{K_{C}n}{{\gamma V}_{\mathrm{RBC}}V_{\mathrm{BUFFER}}}$$

(7) $${a=K}_{C}\;\frac{{\gamma V}_{\mathrm{RBC}}{+V}_{\mathrm{BUFFER}}}{{\gamma V}_{\mathrm{RBC}}V_{\mathrm{BUFFER}}}$$

and therefore:

(8) $$\frac{b}{a}=\frac{n}{{\gamma V}_{\mathrm{RBC}}{+V}_{\mathrm{BUFFER}}}$$

which is the equilibrated concentration (C_BUFFER (eq)_). These equations are comparable to those as derived previously, with the difference of γ [23].

Intercompartment clearance K_C_ is determined by erythrocyte volume (i.e., erythrocyte suspension volume, V_susp_, times hematocrit, H_susp_) and k_s_ (1/min):

(9) $$K_{C}{=k}_{s}{\;H}_{\mathrm{susp}}{\;V}_{\mathrm{susp}}$$

where, k_s_ represents the specific rate constant, which is a more general measure for the diffusion rate across the erythrocyte membrane. For this parameter, the hematocrit (H_susp_ = V_RBC_/(V_RBC_ + V_BUFFER_)) and water fractions for BUFFER (f_BUFFER_ = V_BUFFER, w_/V_BUFFER_) and erythrocyte (f_RBC_ = V_RBC, w_/V_RBC_)) compartments are introduced because blood is usually measured as bulk volume:

(10) $$k_{s}=\frac{a}{\frac{1}{f_{\mathrm{BUFFER}}}\;\left(\frac{H_{\mathrm{susp}}}{{1-H}_{\mathrm{susp}}}\right)+\frac{1}{{\gamma \;f}_{\mathrm{RBC}}}}$$

In loading experiments without an inhibitor, the model parameters k_s_ and γ were identified by fitting the function in Equation (5) and the macro parameters a (Equation (7)) and C_BUFFER(eq)_ (= b/a, Equation (8)) to experimental data: C_BUFFER(t)_, H_susp_, mass of the erythrocyte suspension and concentration as well as volume of the added PBUT mix. In loading experiments in the presence of an inhibitor, the model parameter k_s_ was identified according to the same procedure, whereas the individual value for γ was assumed to be the same as in loading experiments without an inhibitor and was therefore fixed for each healthy subject. In unloading experiments, mean values for γ, as obtained in loading experiments without an inhibitor, were used to identify k_s_ and the following experimental data were used to fit the function in Equation (5) and the macro parameters a (Equation (7)) and C_BUFFER(eq)_ (= b/a, Equation (8)): C_BUFFER(t)_, H_susp_, mass of the erythrocyte suspension and the measured PBUT concentration after 1 h (i.e., C_BUFFER(eq)_,m). In each series of experiments, water fractions in plasma and erythrocytes were assumed as f_BUFFER_ = 0.99 and f_RBC_ = 0.70 and Berkeley Madonna software (University of California, Berkeley, CA, USA) was used for parameter identification. The source codes for loading and unloading experiments are provided in Appendix B. Two Berkeley Madonna model files including experimental data, for representative loading and unloading experiments, are available as Appendix B digital materials.

### 5.6. Statistics

Statistical evaluation was performed with GraphPad Prism 4.00 for Windows (GraphPad Software, La Jolla, CA, USA) and data were checked for normality (Shapiro–Wilk test). Normally distributed data are presented as mean ± standard deviation, whereas non-normal data are presented as median (25th, 75th percentile). Either paired t-tests or Wilcoxon signed rank test, Mann–Whitney tests as well as repeated measures ANOVA tests, and Friedman tests with Tukey (ANOVA test) or Dunns (Friedman tests) post hoc analysis were used where appropriate.

## Figures and Tables

**Table 1:** Experimental data for loading and unloading experiments.

Type of Experiment	C_BUFFER(0)_,c (µmol/L)	C_BUFFER(eq)_,m (µmol/L)	H_wb_ (%)	H_susp_ (%)
*Loading (n = 8)*			47 ± 5	46 ± 5
HA	802.9 ± 72.3	504.3 ± 33.1		
IAA	19.2 ± 1.7	9.2 ± 0.5		
IS	227.6 ± 20.5	92.5 ± 7.0		
*p*CS	209.6 ± 18.9	122.9 ± 13.1		
*Loading + inhibitor (n = 8)*			47 ± 5	46 ± 5
HA	804.3 ± 76.4	518.6 ± 42.4		
IAA	19.2 ± 1.8	8.3 ± 0.5 *		
IS	228.0 ± 21.7	146.5 ± 14.5 *		
*p*CS	210.0 ± 20.0	131.8 ± 12.5		
*Unloading (n = 6)*			36 ± 3	32 ± 3
HA	0	48.6 (32.6;52.4)		
IAA	0	1.08 (0.66;1.91)		
IS	0	10.5 (6.76;12.5)		
*p*CS	0	40.4 (25.8;82.5)		
*Unloading + inhibitor (n = 6)*			36 ± 3	33 ± 3
HA	0	40.2 (28.8;43.9) *		
IAA	0	0.84 (0.47;1.37) *		
IS	0	5.55 (4.05;7.71) *		
*p*CS	0	33.8 (16.7;61.3) *		

HA: hippuric acid; IAA: indole-3-acetic acid; IS: indoxyl sulfate; *p*CS: *p*-cresyl sulfate; C_BUFFER(0)_, c: calculated PBUT concentration in the buffer at t = 0 min; C_BUFFER(eq)_,m: measured PBUT concentration in the buffer at equilibrium; H_wb_: hematocrit in native whole-blood sample; H_susp_: hematocrit in erythrocyte suspension. Values are mean ± standard deviation or median (25th percentile (pct); 75th pct). *p < 0.05 between experiments with and without an inhibitor; *p* < 0.05 for all solutes between start and equilibrium concentration.

**Table 2:** Kinetic data for loading and unloading experiments.

Type of Experiment	a (1/min)	k_s_ (1/min)	K_C_ (mL/min)	γ
*Loading (n = 8)*				
HA	0.06 ± 0.02	0.03 ± 0.01	0.16 ± 0.04	1.24 ± 0.20
IAA	1.46 ± 0.02^a^	0.82 ± 0.10^a^	4.66 ± 0.23^a^	1.54 ± 0.13^a^
IS	0.27 ± 0.07^b^	0.19 ± 0.05^b^	1.10 ± 0.27^b^	2.72 ± 0.39^a,b^
*p*CS	0.85 ± 0.56^a,b,c^	0.40 ± 0.23^a,b,c^	2.38 ± 1.46^a,b,c^	1.30 ± 0.24^b,c^
*Loading + inhibitor (n = 8)*				
HA	0.04 ± 0.01^d^	0.02 ± 0.01^d^	0.12 ± 0.04^d^	1.24 ± 0.20^1^
IAA	1.47 ± 0.001^a^	0.83 ± 0.10^a^	4.68 ± 0.19^a^	1.54 ± 0.13^1,a^
IS	0.03 ± 0.004^a,d^	0.02 ± 0.003^b,d^	0.10 ± 0.01^b,d^	2.72 ± 0.39^1,a,b^
*p*CS	0.05 ± 0.007^b,c,d^	0.02 ± 0.004^b,d^	0.13 ± 0.02^b,d^	1.30 ± 0.24^1,b,c^
*Unloading (n = 6)*				
HA	0.10 (0.08;0.13)^e^	0.06 (0.05;0.08)^e^	0.38 (0.33;0.52)^e^	1.24^2^
IAA	0.51 (0.39;3.29)^a^	0.35 (0.28;2.39)^a^	2.36 (1.58;15.0)^a^	1.54^2^
IS	0.39 (0.35;1.30)^a,e^	0.38 (0.34;1.33)^a,e^	2.53 (1.98;8.40)^a,e^	2.72^2^
*p*CS	0.48 (0.40;2.28)^a,e^	0.29 (0.25;1.46)^a,b,e^	2.00 (1.42;9.31)^a,b,e^	1.30^2^
*Unloading + inhibitor (n = 6)*				
HA	0.07 (0.05;0.09)^e^	0.04 (0.03;0.06)^e^	0.25 (0.21;0.36)^e^	1.24^2^
IAA	2.19 (0.71;3.66)^a^	1.52 (0.50;2.61)^a^	8.79 (3.21;16.5)^a^	1.54^2^
IS	0.77 (0.43;3.35)^a,e^	0.73 (0.43;3.49)^a,e^	4.93 (2.74;19.7)^a,e^	2.72^2^
*p*CS	2.08 (0.45;4.18)^a,c,e^	1.35 (0.28;2.62)^a,e^	8.03 (1.83;15.0)^a,e^	1.30^2^

HA: hippuric acid; IAA: indole-3-acetic acid; IS: indoxyl sulfate; *p*CS: *p*-cresyl sulfate; a: equilibration time constant; k_s_: specific rate constant; K_C_: intercompartment clearance; γ: solute partition coefficient. Values are mean ± standard deviation or median (25th pct; 75th pct).^a, b, or c^ *p* < 0.05 versus HA, IAA, or IS respectively, as obtained within the same series of experiments.^d^ *p* < 0.05 versus corresponding experiments without the inhibitor.^e^ *p* < 0.05 versus corresponding loading experiment.^1^ or ^2^ Individual respectively, mean values for γ, as obtained in loading experiments without the inhibitor, were used to fit parameters.

**Figure A1:**
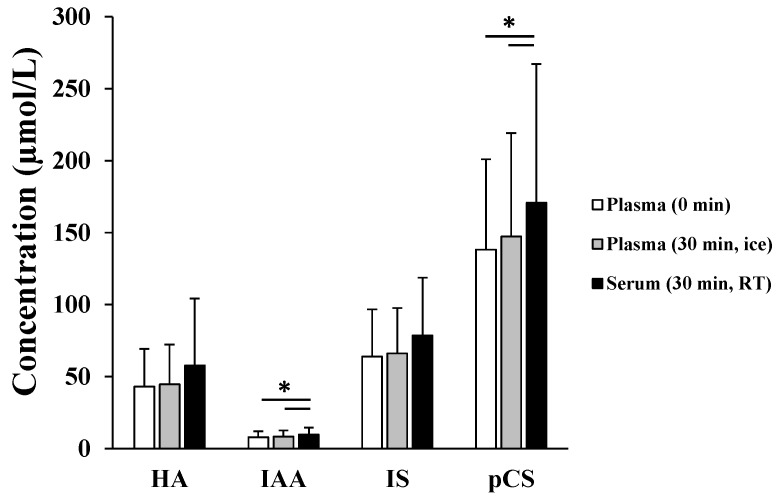
Effect of time between sampling and blood centrifugation on the serum and plasma concentrations of hippuric acid (HA), indole-3-acetic acid (IAA), indoxyl sulfate (IS), and *p*-cresyl sulfate (*p*CS) as determined in blood samples collected at the dialyzer outlet. Blood tubes were centrifuged either immediately (plasma (0 min)), after 30 min on ice (plasma (30 min, ice)), or after 30 min at room temperature (serum 30 min, RT). * *p* < 0.05.

## Supplementary Materials

The following are available online at https://www.mdpi.com/2072-6651/11/7/385/s1, Supplementary File 1: HA_Healthy.txt, Supplementary File 2: HA_HDPatient.txt.

## Appendix A

Extra blood samples from 10 stable HD patients were collected at the dialyzer outlet line after 120 min of dialysis (performed according to the Declarations of Helsinki, approved by the Ethics Committee of Ghent University Hospital (2017/0162) and all participants gave their written informed consent). Blood was collected in two different K_2_EDTA plasma tubes and in serum separating tubes (all Becton Dickinson Company, NJ, USA). Plasma tubes were centrifuged either immediately after sampling or after a period of 30 min on ice, whereas serum tubes were left at room temperature for 30 min to allow for clotting. Total serum and plasma PBUT concentrations were determined by UHPLC-UV/FLD [36]. Statistical analysis was performed with GraphPad Prism 4.00 for Windows (GraphPad Software, La Jolla, CA, USA) and data were checked for normality (Shapiro–Wilk test). Repeated measures ANOVA and Friedman tests with, respectively, Tukey and Dunns post hoc analysis, were used when appropriate, *p* < 0.05 was considered significant.

Serum concentrations were significantly increased for IAA and *p*CS (Figure A1), while only a visual trend was observed for HA and IS in blood samples allowed to clot within 30 min at room temperature, as compared to plasma samples immediately separated after collection. Placing blood samples on ice for 30 min attenuated the equilibration process resulting in only a small increase in extracellular concentration (trend only).

These results indicate that when blood samples are collected the moment there is a disequilibrium between intra- and extra-cellular concentrations (e.g., in samples taken at the dialyzer outlet line or in samples taken at a later stage during dialysis), plasma concentrations will depend on the time between sampling and centrifugation as well as on the solute equilibration rate. Because immediate centrifugation after collection is not always achievable in clinical practice, this process of solute equilibration can be attenuated by placing blood samples immediately on ice prior to centrifugation.

## Appendix B

### Appendix B.1. Berkeley Madonna Script for Loading Experiments for Hippuric Acid (HA) without Inhibitor

{Identification of model parameters ks and gamma from equilibration in solute loading tests using HA data from “HA_Healthy.txt” file using the exact analytical solution and Berkeley-Madonna version 8.3 or 9.1 software (https://berkeley-madonna.myshopify.com).

Open a new file from the “File” dropdown menu and delete any default information from the opening window.

Copy and paste the source code (from the first “{“ to the last “}” of this text from the on-line full text html-document as plain TEXT into that window.

Load the experimental sample data from the “Model” drop-down menu using the “Datasets” command.

Import the “HA_Healthy.txt” data (Supplementary File 1) as 1D vector.

Run (click the “RUN” icon) this model and plot the data vs time.

Double-click the figure and select the data variable for display.

Then use “Curve fit” in the “Parameter” drop-down menu, select the parameters “a” and “gamma”, and press “o.k.”

The model “ct” is fit to experimental data.

The parameters identified from the optimal fit can be read in the “parameter” window or by clicking the “P” icon in the plot.

The numerical values for “ks” and “Kc” and selected variables can be displayed by switching from “plot-view” to “table view”}

STARTTIME = 0

STOPTIME = 70

DT = 0.02

Hsusp = 0.425; hematocrit of erythrocyte suspension

Msusp = 13.05; mass of erythrocyte suspension in g

Cs = 82603; concentration of HA in PBUT mix in µmol/L

Vs = 0.000065; volume of spiking solution in L

fBUFFER = 0.99; water fraction in BUFFER

fRBC = 0.70; water fraction in erythrocytes

rhosusp = 1050; erythrocyte suspension density in g/L

a = 0.06; exponent, slope of the experimental decrease

gamma = 1; solute partition coefficient

DISPLAY ct, a, ks, Kc, gamma

Ct = (c0-ceq) * exp(-a * TIME) + ceq; BUFFER concentration at time t in µmol/L

c0 = ntot/(Vsusp * (1-Hsusp) * fBUFFER + Vs); initial BUFFER concentration in µmol/L

ceq = ntot/(Vsusp * (1-Hsusp) * fBUFFER + Vsusp * Hsusp * fRBC * gamma + Vs)

{BUFFER concentration at equilibrium in µmol/L}

Ks = a/(Hsusp/(1-Hsusp)/fBUFFER + 1/(gamma * fRBC)); specific rate constant in 1/min

Kc = ks * Hsusp * Vsusp * 1000; intercompartment clearance in mL/min

Vsusp = Msusp/rhosusp; volume of erythrocyte sample in L

Ntot = cs * Vs; total mole of solute in erythrocyte suspension in µmol {End of script}

### Appendix B.2. Berkeley Madonna Script for Unloading Experiments for Hippuric Acid (HA)

{Identification of model parameter “ks” from equilibration in solute unloading tests with experimental HA data from “HA_HDPatient.txt” data file using the exact analytical solution and Berkeley-Madonna version 8.3 to 9.1 software (https://berkeley-madonna.myshopify.com).

Open a new file from the “File” drop-down menu and delete any default information from the opening window.

Copy and paste the source code (from the first “{“ to the last “}” of this text from the on-line full text html-document as plain TEXT into that window.

Load the experimental sample data from the “Model” drop-down menu using the “Datasets” command.

Import the “HA_HDPatient.txt” data (Supplementary File 2) as 1D vector.

Run (click the “RUN” icon) this model and plot the data vs. time.

Double-click the figure and select the data variable for display.

Then use “Curve fit” in the “Parameter” drop-down menu, select parameters “a” and “ceq” and press “o.k.”.

The model is fit to experimental data.

The parameters identified from the optimal fit can be read in the “Parameter” window or by clicking the “P” icon in the plot.

The numerical values for “ks” and “Kc” and selected variables can be displayed by switching from “plot-view” to “table view”}

STARTTIME = 0

STOPTIME = 70

DT = 0.02

Hsusp = 0.32; hematocrit of erythrocyte suspension

Msusp = 13.27; mass of erythrocyte suspension in g

Ceq = 70.24; measured BUFFER concentration at equilibrium after 1 h in µmol/L

c0 = 0; solute concentration in BUFFER at t = 0 in µmol/L

fBUFFER = 0.99; water fraction in BUFFER

fRBC = 0.70; water fraction in erythrocytes

rhosusp = 1050; erythrocyte suspension density in g/L

a = 0.06; exponent, slope of the experimental decrease

gamma = 1.24; solute partition coefficient from loading experiments

DISPLAY ct, ceq, a, ks, Kc, gamma

Ct = (c0-ceq) * exp(-a * TIME) + ceq; BUFFER concentration at time t in µmol/L

Ks = a/(Hsusp/(1-Hsusp)/fBUFFER + 1/(gamma * fRBC)); specific rate constant in 1/min

Kc = ks * Hsusp * Vsusp * 1000; intercompartment clearance in mL/min

Vsusp = Msusp/rhosusp; volume of erythrocyte sample in L {End of script}
